# Balance Impairment in the Burn Population: A Burn Model System National Database Study

**DOI:** 10.3390/ebj5030023

**Published:** 2024-07-25

**Authors:** Edward Santos, Kaitlyn L. Chacon, Lauren J. Shepler, Kara A. McMullen, Mary D. Slavin, Marc van de Rijn, Karen J. Kowalske, Colleen M. Ryan, Jeffrey C. Schneider

**Affiliations:** 1 Department of Physical Medicine and Rehabilitation, Schoen Adams Research Institute, Spaulding Rehabilitation Hospital, Harvard Medical School, Boston, MA 02129, USA; esantos16@mgb.org (E.S.); klchacon@mgb.org (K.L.C.); lshepler@mgb.org (L.J.S.); mvanderijn@mgh.harvard.edu (M.v.d.R.); 2 Department of Rehabilitation Medicine, University of Washington, Seattle, WA 98104, USA; mcmulk@uw.edu; 3 Department of Health Law, Policy, and Management, Boston University School of Public Health, Boston, MA 02118, USA; mslavin@bu.edu; 4 Rehabilitation Outcomes Center at Spaulding, Spaulding Rehabilitation Hospital, Boston, MA 02129, USA; 5 Department of Physical Medicine and Rehabilitation, University of Texas Southwestern Medical Center, Dallas, TX 753890, USA; karen.kowalske@utsouthwestern.edu; 6 Department of Surgery, Massachusetts General Hospital, Harvard Medical School, Boston, MA 02114, USA; cryan@mgh.harvard.edu; 7 Shriners Hospitals for Children, Harvard Medical School, Boston, MA 02114, USA

**Keywords:** burns, inpatient rehabilitation, balance, outcomes research

## Abstract

Balance is an important component of daily function and impairments can lead to injury and quality-of-life limitations. Balance is not well studied in the burn population. This study examines the frequency of long-term balance impairments and associated factors after a burn injury. The Burn Model System National Database was analyzed. Trouble with balance was self-reported at discharge, 6, 12, 24, and 60 months after injury. Regression analyses examined the associations between demographic and clinical characteristics and balance impairments at 12 months. Of 572 participants, balance impairments were most reported at discharge (40.3%), continuing over 60 months (26.8–36.0%). Those reporting balance impairments (*n* = 153) were more likely to be older, unemployed, have Medicaid or Medicare, receive inpatient rehabilitation, receive outpatient physical or occupational therapy, have vision problems, have leg or feet burns and swelling, and have foot numbness compared to those without (*p* ≤ 0.001). Regression analysis demonstrated a 4% increased odds of balance impairment for every increase in year of age (*p* < 0.001), 71% lower odds if employed at time of injury (*p* < 0.001), and 140% higher odds if receiving outpatient physical or occupational therapy at 12 months (*p* = 0.008). Common reports of balance impairments highlight the need for routine screenings to identify burn survivors that may benefit from targeted interventions.

## 1. Introduction

The word “balance” in English comes from the Latin word (*libra*) *bilanx* that means two (bi-) scale pans (-lanx) [1], connecting to the idea of a scale or balance. Balance is a key part of our health, longevity, and performance in activities of daily living [2,3]. Balance involves many intrinsic and extrinsic factors and is affected by various systems in the body including visual, vestibular, proprioception, somatosensory, and auditory [4,5]. Declines in balance occur with aging-related changes, leading to potential falls and injury [2,3]. In addition, there are many factors that can make an individual susceptible to falls such as other chronic conditions, polypharmacy, socioeconomic status, environmental risks (poor footwear, tripping hazards, lack of handrails, poor lighting within or outside their home, living alone), and poor safety and accessibility [6,7,8,9,10]. Among older adults, falls are often responsible for injuries and are a marker of disease severity [11].

Over the last 30 years, burn injury survival rates have continued to improve due to advancements in the standards of care [12]. However, individuals living with a burn injury continue to face a myriad of physical and psychosocial challenges years after injury [13]. Of these challenges, trouble with balance was reported two years after injury in approximately one-quarter of participants in a longitudinal study of burn outcomes [14]. Various burn injury sequelae may contribute to impaired balance, including altered sensory perception [15], debilitation, contractures, nutritional deficiencies, pain, and neuropathies [16]. Preexisting factors may also contribute to balance impairment and fall risk such as vestibular problems, joint or muscle problems, low vision or vision impairment, muscle mass loss, low blood pressure, and other comorbidities (such as diabetes, heart disease, stroke, Parkinson’s, thyroid problems, and other neurological conditions) [17,18,19,20,21].

While previous literature has examined interventions for balance performance and control [22,23] the prevalence of balance impairment and associated risk factors in the burn population is underexplored in the literature [16]. Therefore, the purpose of this study is to describe the frequency in which burn survivors report balance impairments long term and determine the associated risk factors. 

## 2. Materials and Methods

### 2.1. Burn Model System National Database

This study used data from the Burn Model System (BMS) National Database, funded by the National Institute on Disability, Independent Living, and Rehabilitation Research. This study was approved by the Mass General Brigham Institutional Review Board (#2011P001264) as part of the overarching BMS program. The BMS Database was established in 1994 as a means of exploring the long-term sociodemographic, clinical, and patient reported outcomes of data of burn survivors. Written informed consent was obtained from all participants and the Institutional Review Board at each BMS site oversaw data collection [24]. Data were requested from the BMS National Data and Statistical Center through their standard operating procedure: (https://burndata.washington.edu/sites/burndata/files/files/602BMS_InternalNotificationSOP_2024-2-28.pdf) (accessed on 1 December 2022). Participants who had a burn injury between 1 August 2015 and 30 January 2023, who speak and understand English and/or Spanish, and were consented and alive at discharge were included in this study. During the study period, the following four sites were included: Boston-Harvard Burn Injury Model System (BH-BIMS), Boston, MA, USA; North Texas Burn Rehabilitation Model System, Dallas, TX, USA; Northwest Regional Burn Model System, Seattle, WA, USA; and the Pediatric Burn Injury Rehabilitation Model System, Galveston, TX, USA. Database eligibility included adults with a burn injury that required autografting or amputation surgery for wound closure and met one of the following criteria: (1) 18–64 years of age with a burn injury ≥ 20% total body surface area (TBSA); (2) ≥65 years of age with a burn injury ≥ 10% TBSA; (3) any age with a burn injury to their critical areas including face/neck, hands, or feet; and (4) any age with a high-voltage electrical burn injury. BMS Database enrollment criteria have been modified over time and the data sets generated during and/or analyzed during the current study are publicly available upon request from the BMS National Data and Statistical Center’s website: https://burndata.washington.edu (accessed on 30 January 2023) [24]. 

### 2.2. Demographic and Clinical Characteristics

Demographic and clinical variables were obtained through self-report or medical record abstraction. The following demographic variables were examined: age, sex, highest level of education, ethnicity, race, employment status, and insurance type. Clinical variables collected were BMS site (intentionally deidentified), burn etiology, lower extremity amputation (Y/N), alcohol and drug misuse (both variables from the Cut down, Annoyed, Guilty, and Eye-opener (CAGE) Scale; the CAGE Scale is considered positive if the score is greater than or equal to 2; preinjury recall collected at discharge) [25], documented range of motion deficits (Y/N), inpatient rehabilitation stay (Y/N), multiple trips to the operating room (≥2; Y/N), burn location (feet/legs; head/neck/face), vision problems (Y/N), numbness in feet (Y/N), swollen feet or legs (Y/N), comorbidities (diabetes, stroke, Parkinson’s disease, spinal cord injury, and traumatic brain injury; Y/N), burn size (percent TBSA), body mass index (BMI), prescription pain medication use (Y/N), and outpatient physical or occupational therapy received (Y/N). All the demographic and clinical variables were collected at discharge, except for outpatient therapy and prescription pain medication use, which were both collected at the 12-month follow-up. 

### 2.3. Primary Outcomes

The primary outcome was the item, “Trouble with your balance?” (yes/no) in the Adult Review of Systems section of the BMS follow-up questionnaire at the 12-month timepoint. This item is also collected at discharge and at 6, 24, and 60 months after a burn injury. The 12-month timepoint was chosen a priori by the authors because long-term balance outcomes are more visible. Additionally, this timepoint ensured a more robust sample size given documented higher participant attrition rates at more distal timepoints [26].

### 2.4. Data Analysis

For purposes of analysis, the study population was divided into the following two groups: those with and those without self-reported balance impairments at 12 months. Differences in demographic and clinical variables between groups were tested using nonparametric tests (Wilcoxon–Mann–Whitney) due to the nonnormality of the data for continuous variables and chi-square tests for Fisher’s exact for categorical variables. A *p*-value less than 0.002 was considered significant, using the Bonferroni method to adjust for multiple comparisons.

The number of participants who reported balance impairments at discharge and all follow-up timepoints were analyzed. Additionally, the frequency of balance impairments at discharge and follow-up (6, 12, 24, and 60 months) among those with repeated measures was examined. For these repeated measures analyses, pairwise deletion was used for those with missing data on any timepoint. Balance impairments were also assessed by age category (18–30, 31–40, 41–50, etc.). Statistically significant differences in the frequency of balance impairments by age categories were assessed using an omnibus chi-square statistical test. 

Logistic regressions were used to examine the association between demographic and clinical characteristics and self-reported balance impairments (1 = yes, 0 = no) at 12 months postinjury. The dependent variables included age, sex, BMS site, BMI, lower limb amputation, employment at the time of injury, inpatient rehabilitation stay, multiple trips to the operating room, education (greater than high school vs. high school and less), alcohol and drug misuse, and therapy services. Additional considered variables were not included in the models due to missing data (detailed in the Results Section). All included variables were collected at discharge with the exception of therapy services (12 months). A *p*-value less than 0.05 was considered significant. Robust standard errors accounted for heteroskedasticity; interactions were examined, and model fit was checked. Additionally, an exploratory regression analysis examined demographic and clinical variables associated with improvement in self-reported balance from discharge to 12 months. Stata 15.1 was used for all analyses [27]. 

## 3. Results

### 3.1. Descriptive Analysis

A total of 572 BMS Database participants met the inclusion criteria for this study, with 153 reporting trouble with balance and 419 reporting no trouble with balance at 12 months after injury (Table 1). 

Compared to those without balance impairments, the balance impairment group was older (median 53.3 (interquartile range: 42.1, 63.4) vs. 44.7 (31.1, 57.0), *p* < 0.001), less likely to be employed at the time of injury (49.0% vs. 74.2%, *p* < 0.001), and more likely to have Medicaid/Medicare insurance (22.7%/31.3% versus 12.7%/15.1%, *p* < 0.001), an inpatient rehabilitation stay (34.7% vs. 21.3%, *p* = 0.001), outpatient physical or occupational therapy (41.7% vs. 23.7%, *p* < 0.001), vision problems (26.2% vs. 7.0%; *p* < 0.001), leg or feet burns (79.8% vs. 62.1%; *p* < 0.001), numbness in their feet (55.7% vs. 16.8%, *p* < 0.001), and swollen feet or legs (46.3% vs. 14.9%, *p* < 0.001). 

The variables not statistically different between groups were sex, education, ethnicity, race, BMS site, burn etiology, lower extremity amputation, alcohol misuse, drug misuse, range of motion deficit, multiple trips to the operating room, head/neck/face burn, TBSA, and BMI. 

The following variables had significant missing data (61–89%) and, therefore, were not included in the analysis: prescription pain medication use at 12 months and comorbidities at discharge (diabetes, stroke, Parkinson’s, spinal cord injury, traumatic brain injury). These variables were missing data due to the timeframe of data collection (i.e., item collection began after 2015) or missing data in the medical record at follow-up timepoints. 

### 3.2. Primary Outcomes over Time

Balance impairments were most frequently reported at discharge (40.3%) and ranged from 26.8% to 36.0% over the five-year follow-up period after injury (Table 2). It is important to note that this data in Table 2 is cross-sectional leading to different sample sizes at each timepoint and excludes participants whose windows for data collection were not yet open (e.g., the five-year datapoint excludes participants with a burn injury after 2019).

The frequency of trouble with balance for participants with repeated measures was examined at discharge and all follow-up timepoints (Table 3). In all groups with repeated balance measures, a similar percentage of participants reported impairments of balance at discharge (40.0–51.8%) and at least one timepoint for follow-up (26.3–35.2%). 

### 3.3. Association between Age and Self-Reported Balance Impairments

**Figure 1:**
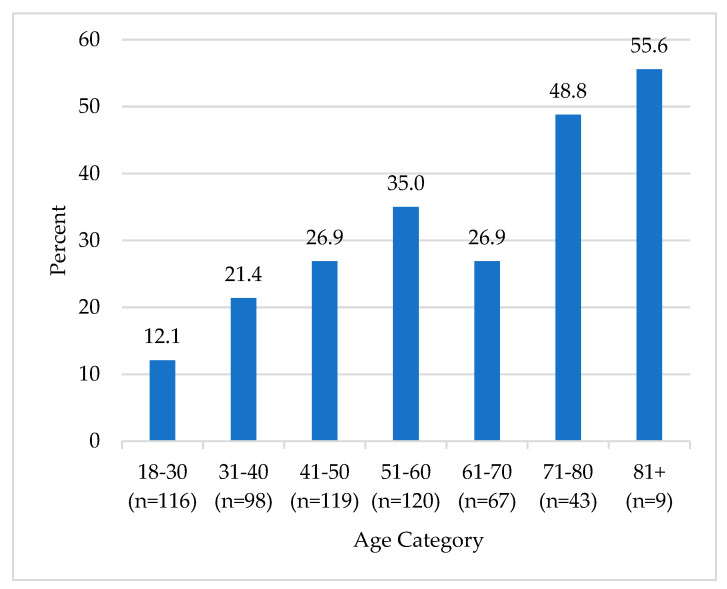
Histogram of the association between age and self–reported balance impairments. Differences were examined using the omnibus (chi-square) test, *p* < 0.001.

An examination of the relationship between self-reported balance impairments and age demonstrated that trouble with balance was increasingly common with older age (*p* < 0.001; Figure 1).

### 3.4. Regression Analysis

Logistic regression analysis examined the association between self-reported trouble with balance at 12 months and demographic and clinical factors (Table 4).

For every increase in one year of age, there is a 4% increased odds of a balance impairment (*p* < 0.001). Participants who were employed at the time of injury had a 71% lower odds of reporting a balance impairment (*p* < 0.001). Trouble with balance was 140% higher for participants who received outpatient physical or occupational therapy at 12 months (*p* = 0.008). Interaction between inpatient rehabilitation and outpatient physical or occupational therapy services was not significant. 

An exploratory regression analysis examined the associations with improvement in balance and demonstrated that employment prior to a burn injury is associated with a 617% higher odds of improved balance from discharge to 12 months (*p* = 0.001) compared to those not employed prior to injury. Participants who received outpatient physical or occupational therapy services at 12 months exhibited a 72% lower odds of reporting balance improvement from discharge to 12 months (*p* = 0.017).

## 4. Discussion

The frequency of balance impairments and its association with clinical and demo-graphic variables has been underexplored in the burn population; thus, this study helps inform an important gap in the existing literature. This study found that balance impairments are commonly reported at discharge (40.3%) and can persist for up to five years after injury (26.8–36.0%). Those with balance impairments were more likely to be older, unemployed, have Medicaid or Medicare, receive inpatient rehabilitation, receive outpatient physical or occupational therapy, have vision problems, have leg or feet burns and swelling, and have foot numbness compared to those without balance impairments. The regression analysis demonstrated that balance impairments are more likely in people living with a burn injury who are older, were unemployed at the time of injury, and received outpatient physical or occupational therapy. 

Consistent with the general balance literature, balance impairments in people living with a burn injury are more common with older age. A prior study demonstrated a significant association between age and balance disorders with the prevalence of balance impairments increasing with age [28]. Trouble with balance is common in the US elderly population, with 34% of elderly individuals reporting a decline in balance and 23% reporting a fall in the prior 12 months [29]. Using an epidemiologic study of self-reported balance disorders in US adults as a comparison, this study found higher percentages of burn survivors reporting balance impairments across all similar age categories [30]. Due to the elderly being the fastest growing portion of the global population [31] and burn survivors living longer due to advancements in care [32], age is a key factor to consider when planning prevention measures for elderly individuals living with a burn injury. Additionally, various environmental factors, which are not contained in the BMS database, may influence elderly individuals’ fall risk, including availability of supportive and accessible living facilities, adaptive equipment, and social support resources. Many of these environmental supports come at a financial cost to the individual, highlighting societal inequities to environmental supports [33].

There is some evidence from this study that unemployment prior to injury could be associated with balance impairments after a burn. This is crucial because employment is identified as a social determinant of health and unemployment is historically tied to poorer health outcomes [34,35]. Full-time employment has predicted significantly slower declines in perceived health and physical functioning, compared to unemployment [36]. Specifically in women, employment is associated with a reduced likelihood of physical function impairments [37]; those who work part time or are unemployed are more likely to experience a physical impairment [37]. Also, there is an association between time spent not working and a likelihood of experiencing a severe physical functional impairment [37]. Additionally, employment confers a protective effect on hospitalization in older adults over their lifetime; in contrast, having an illness or disability is a predictor of hospitalization later in life [38]. In burns, return to work is an important milestone for survivors. The relationship between preburn and postburn employment and postburn balance is complex and the directional relationship over time is hard to interpret. With that in mind, some studies have found that being employed preinjury is highly associated with being employed postinjury [39,40] and that unemployment has been tied to poorer health outcomes [41]. It is worth noting that not-working retired individuals are more likely to be older and therefore also have worse balance due to their age.

In this study, receiving outpatient physical or occupational therapy is associated with balance impairments after a burn injury. Both physical and occupational therapies have been found to be beneficial in improving balance functionality [42]. This is important because individuals who received therapy services for trouble with balance are likely to require these services to help manage their functional deficits. In a study with elderly individuals with fall risk, those who received a combination of rehabilitation services at home (such as physical, occupational, speech therapies, or nursing interventions) were found to have an overall positive change in gait and balance, compared to just physical therapy alone [43]. In another study of elderly over 75 years of age with trouble with balance, individuals who received a physical therapy intervention had a greater improvement in balance at one month compared to a control group; however, the effect was not detected at one-year follow-up [44]. These prior works suggest that individuals living with chronic conditions that affect balance may benefit from long-term outpatient therapy services.

### Limitations

There were several limitations to this study. Some clinical factors that may contribute to balance impairments are not collected as part of the BMS database, including escharotomy, fasciotomy, reasons for ongoing physical and occupational therapy, and environmental factors (e.g., delays in provision of adaptive aids, equipment, or home modifications delays). The database includes PROMIS Pain Intensity and Interference and collects pain medication use (a lot of missing data) but does not include the cause of ongoing pain or peripheral neuropathies. In addition, prescription pain medication uses at 12 months and comorbidities at discharge (diabetes, stroke, Parkinson’s, spinal cord injury, traumatic brain injury) were not included in the regression analysis due to a substantial amount of missing data. There is increasing missing data at more distal follow-up timepoints. A potential sample bias is that the BMS Database includes participants with more severe burn injuries [45]. Additionally, there is a selection (did not enroll) and survey response bias (failed to respond, unable to locate, preferred to not respond to item). Prior examination of the BMS Database found that participants who were unemployed, did not have private health insurance or workers’ compensation insurance, and had a history of drug abuse exhibited an increased risk of loss to follow-up across two or more timepoints [26]. Also, participants are less likely to respond at their next follow-up timepoint if they missed the previous follow-up [45]. Although the BMS has been found to be limited in its representation of diverse populations [46], nevertheless, the database has been found to be representative of the general US population [45].

## 5. Conclusions

This study found that balance impairments are common (40.3% at discharge) and can persist for up to five years after injury (36.0% at 60 months). Furthermore, this study examined data up to the five-year follow-up timepoint since this is the specified interval of data collection in the database; however, future studies are needed to examine the presence of balance impairments beyond five years and the contribution of factors not collected in the database such as environmental factors. Additionally, factors such as age, employment status prior to injury, and outpatient therapy services were associated with balance impairments in the burn population. Although not previously well reported in the literature, data from this study found that balance impairments in the burn population are prevalent and this study may inform future use of balance assessments and intervention in follow-up care. Future clinical recommendations should include (1) screening for fall risk during clinical encounters including acute hospitalization, inpatient rehabilitation, and at follow-up burn and primary care appointments; (2) targeted balance therapy for inpatients; and (3) outpatient therapy referrals for individuals with an elevated fall risk. Further research is needed to examine the effectiveness of balance assessment and potential interventions in the burn population.

## Figures and Tables

**Table 1:** Comparison of populations with and without balance impairments at 12 months (*n* = 572).

Variable	With Balance Impairments (*n* = 153)	Without Balance Impairments (*n* = 419)	*p*-Value
Age, median years (IQR)	53.3 (42.1, 63.4)	44.7 (31.1, 57.0)	<0.001 *
Male, *n* (%)	99 (64.7)	293 (69.9)	0.234
Higher than high school education, *n* (%)	65 (51.6)	194 (53.9)	0.656
Hispanic/Latino ethnicity, *n* (%)	22 (14.8)	91 (22.6)	0.043
Race, *n* (%)			
White	121 (80.7)	348 (85.9)	0.007
African American/Black	22 (14.7)	25 (6.2)	
Other ^ꝉ^	7 (4.7)	32 (7.9)	
Employed at time of injury, *n* (%)	70 (49.0)	296 (74.2)	<0.001 *
Insurance ^¥^, *n* (%)			
Medicaid	34 (22.7)	53 (12.7)	<0.001 *
Medicare	47 (31.3)	63 (15.1)	
Private	36 (24.0)	163 (39.1)	
Other ^ꝉ^	15 (10.0)	75 (18.0)	
No insurance	18 (12.0)	63 (15.1)	
Burn Model System Site, *n* (%)			
Site A	31.4 (48)	35.3 (148)	0.419
Site B	34.6 (53)	28.2 (118)	
Site C	34.0 (52)	35.8 (150)	
Burn Etiology, *n* (%)			
Flame	92 (60.1)	231 (55.3)	0.582
Electric	10 (6.5)	31 (7.4)	
Other ^ǂ^	51 (33.3)	156 (37.3)	
Lower extremity amputation, *n* (%)	5 (3.5)	3 (0.8)	0.019
History of alcohol misuse, *n* (%)	21 (14.9)	48 (12.2)	0.416
History of drug misuse, *n* (%)	7 (5.0)	18 (4.7)	0.896
Range of motion deficit, *n* (%)	99 (66.4)	231 (56.1)	0.027
Inpatient rehabilitation, *n* (%)	51 (34.7)	89 (21.3)	0.001 *
Outpatient PT/OT ^§^, *n* (%)	58 (41.7)	93 (23.7)	<0.001 *
Multiple trips to the OR ^¶^, *n* (%)	95 (62.1)	205 (48.9)	0.005
Head/neck/face burn, *n* (%)	79 (51.6)	199 (47.6)	0.394
Feet/leg burn, *n* (%)	122 (79.8)	260 (62.1)	<0.001 *
Vision problems, *n* (%)	39 (26.2)	29 (7.0)	<0.001 *
Numbness in feet, *n* (%)	83 (55.7)	70 (16.8)	<0.001 *
Swollen feet or legs, *n* (%)	69 (46.3)	62 (14.9)	<0.001 *
Burn size, median TBSA ^ꝉꝉ^ percent (IQR)	14.5 (4.0, 3.0)	10.0 (3.0, 24.6)	0.0349
Body Mass Index at admission, median (IQRs)	28.0 (23.8, 32.4)	27.1 (24.2, 31.3)	0.4182

Differences in demographic and clinical characteristics between groups at 12 months were assessed using Wilcoxon–Mann–Whitney tests for continuous variables and chi-square tests for Fisher’s exact for categorical variables. * Significance level was adjusted for multiple comparisons using Bonferroni’s method to 0.002. ^ꝉ^ “Other” Race includes Asian, American Indian/Alaskan Native, Native Hawaiian/Other Pacific Islander, more than one race, some other race, and unknown race; ǂ “Other” Burn Etiology includes scald, contact with hot object, grease, tar, chemical, hydrofluoric acid, radiation, UV light, frostbite, TENS/Stevens Johnson, abrasions, flash burn, necrotizing fasciitis, meningococcemia, and other skin disease; ^§^ Outpatient PT/OT = outpatient physical or occupational therapies; ^¶^ Multiple trips to operating room = two or more during acute hospitalization, collected at discharge; ^ꝉꝉ^ TBSA = total body surface area. ^¥^ The United States has a complex insurance system that consists of insurance types such as Medicaid, Medicare, and private. Medicaid is funded at both the state and federal levels for low-income individuals; Medicare is federally funded for seniors (over 65 in age), and those with disabilities; and lastly, private insurances are acquired individually or through employers with varying coverage and costs. ^ꝉ^ “Other” insurance includes worker’s compensation (L&I), Champus/Tri-Care, self-pay or indigent (public support), VA, or philanthropy (private support or private foundation or Shriners hospital).

**Table 2:** Frequency of balance impairments at up to five years after a burn injury.

Timepoint ^ǂǂ^	Those with Balance Impairments, *n* (%)	Total *n* at Timepoint	Missing Data
Discharge	365 (40.3)	906	156
6 months	194 (28.7)	675	479
12 months	153 (26.8)	572	510
24 months	144 (28.6)	503	445
60 months	58 (36.0)	161	539

^ǂǂ^ Note that this data is cross-sectional leading to different sample sizes at each time point and excludes participants whose windows for data collection were not yet open (e.g., the five-year datapoint excludes participants with a burn injury after 2019).

**Table 3:** Frequency of balance impairments at discharge and each follow-up timepoint for participants with repeated measures.

Follow-Up Timepoint	Response to Balance Item at Discharge and Follow-Up Timepoint, *n*	Balance Impairment at Discharge, *n* (%)	Balance Impairment at Follow-Up Timepoint, *n* (%)	Follow-Up Timepoint	Response to Balance Item at Discharge and Follow-up Timepoint, *n*
6 Months	591	243 (41.2)	161 (27.2)	6 Months	591
12 Months	483	193 (40.0)	127 (26.3)	12 Months	483
24 Months	343	139 (40.5)	102 (29.7)	24 Months	343
60 Months	54	28 (51.8)	19 (35.2)	60 Months	54

Legend: Each row represents a subgroup of the population that responded to the balance item at discharge and the follow-up timepoint indicated in the first column. For example, the first row includes 591 participants who responded to the balance item at both discharge and 6 months. Of those 591 individuals, 243 reported a balance impairment at discharge and 161 at 6 months. Some participants responded to multiple follow-up timepoints; therefore, there is some overlap in the subgroups of each row.

**Table 4:** Logistic regression analysis examining the association between demographic and clinical characteristics and self-reported balance impairments at 12 months.

Variable	Odds Ratio	Robust SE	Z	*p*-Value	95% Cl
Age	1.04	0.01	3.85	<0.001 **	1.02–1.07
Female	0.95	0.33	−0.15	0.881	0.48–1.87
Greater than high school education	0.67	0.21	−1.28	0.201	0.36–1.24
Employed at time of injury	0.29	0.10	−3.50	<0.001 **	0.14–0.58
Burn Model System Site					
Site A	0.98	0.40	−0.06	0.955	0.43–2.19
Site B	0.89	0.36	−0.29	0.768	0.40–1.97
Body Mass Index	0.99	0.02	−0.24	0.813	0.95–1.04
Alcohol misuse at discharge (preinjury recall)	1.44	0.66	0.79	0.432	0.58–3.55
Drug misuse at discharge (preinjury recall)	0.62	0.48	−0.62	0.536	0.14–2.78
Lower limb amputation	3.20	4.71	0.79	0.430	0.18–57.29
Inpatient rehabilitation	1.94	0.77	1.68	0.094	0.89–4.23
Multiple operations ^§§^	1.63	0.60	1.32	0.188	0.79–3.37
Outpatient PT/OT at 12 months ^ꝉꝉꝉ^	2.40	0.79	2.67	0.008 **	1.26–4.57

** Significance level is *p* < 0.05. ^§§^ Multiple trips to the operating room = two or more during acute hospitalization, collected at discharge; ^ꝉꝉꝉ^ Outpatient PT/OT = outpatient physical or occupational therapies.

## Data Availability

The Burn Injury Model System National Database is a prospective, longitudinal, multicenter research data repository that contains measures of functional and psychosocial outcomes following burns. The data are free and publicly available at https://burndata.washington.edu/ (30 January 2023).

## References

[1] Oxford Languages Oxford Dictionaries. Published 2023. https://languages.oup.com/.

[2] Dunsky A. (2019). The Effect of Balance and Coordination Exercises on Quality of Life in Older Adults: A Mini-Review. Front. Aging Neurosci..

[3] Hoogendijk E.O., Afilalo J., Ensrud K.E., Kowal P., Onder G., Fried L.P. (2019). Frailty: Implications for clinical practice and public health. Lancet.

[4] Peterka R.J., Murchison C.F., Parrington L., Fino P.C., King L.A. (2018). Implementation of a Central Sensorimotor Integration Test for Characterization of Human Balance Control During Stance. Front. Neurol..

[5] Rasman B.G., Forbes P.A., Tisserand R., Blouin J.S. (2018). Sensorimotor Manipulations of the Balance Control Loop-Beyond Imposed External Perturbations. Front. Neurol..

[6] Ie K., Chou E., Boyce R.D., Albert S.M. (2021). Fall Risk-Increasing Drugs, Polypharmacy, and Falls Among Low-Income Community-Dwelling Older Adults. Innov. Aging.

[7] Lee H., Lim J.H. (2023). Living Alone, Environmental Hazards, and Falls Among U.S. Older Adults. Innov. Aging.

[8] Li W., Procter-Gray E., Lipsitz L.A., Leveille S.G., Hackman H., Biondolillo M., Hannan M.T. (2014). Utilitarian walking, neighborhood environment, and risk of outdoor falls among older adults. Am. J. Public Health.

[9] Jiang Y., Xia Q., Wang J., Zhou P., Jiang S., Diwan V.K., Xv B. (2019). Environmental risk factors associated with falls among older people living in long-term aged care facilities: A prospective study. Lancet.

[10] World Health Organization, Ageing Life Course Unit (2007). WHO Global Report on Falls Prevention in Older Age.

[11] Cuevas-Trisan R. (2017). Balance Problems and Fall Risks in the Elderly. Phys. Med. Rehabil. Clin. N. Am..

[12] Capek K.D., Sousse L.E., Hundeshagen G., Voigt C.D., Suman O.E., Finnerty C.C., Jennings K., Herndon D.N. (2018). Contemporary Burn Survival. J. Am. Coll. Surg..

[13] Abouzeid C.A., Wolfe A.E., Ni P., Carrougher G.J., Gibran N.S., Hammond F.M., Holavanahalli R., McMullen K.A., Roaten K., Suman O. (2022). Are burns a chronic condition? Examining patient reported outcomes up to 20 years after burn injury-A Burn Model System National Database investigation. J. Trauma Acute Care Surg..

[14] Kelter B.M., Holavanahalli R., Suman O.E., Ryan C.M., Schneider J.C. (2020). Recognizing the long-term sequelae of burns as a chronic medical condition. Burns.

[15] Nedelec B., Hou Q., Sohbi I., Choinière M., Beauregard G., Dykes R.W. (2005). Sensory perception and neuroanatomical structures in normal and grafted skin of burn survivors. Burns.

[16] Schneider J.C., Qu H.D., Lowry J., Walker J., Vitale E., Zona M. (2012). Efficacy of inpatient burn rehabilitation: A prospective pilot study examining range of motion, hand function and balance. Burn.

[17] Mayo Clinic Staff Balance Problems—Symptoms and Causes. Mayo Clinic. Published 2018. https://www.mayoclinic.org/diseases-conditions/balance-problems/symptoms-causes/syc-20350474.

[18] National Institute on Aging Older Adults and Balance Problems. Published 12 September 2022. https://www.nia.nih.gov/health/falls-and-falls-prevention/older-adults-and-balance-problems#causes.

[19] National Institute on Deafness Other Communication Disorders Balance Disorders (NIDCD) Published 12 March 2018. https://www.nidcd.nih.gov/health/balance-disorders.

[20] American Speech-Language-Hearing Association (n.d.) Balance System Disorders (Practice Portal). www.asha.org/Practice-Portal/Clinical-Topics/Balance-System-Disorders/.

[21] Appeadu M., Bordoni B. Falls and Fall Prevention in the Elderly. PubMed. Published 4 June 2023. https://www.ncbi.nlm.nih.gov/books/NBK560761/.

[22] Othman E.M., Toson R.A. (2024). Response of bone mineral density and balance performance in post-burn patients with selected Qigong training: A single-blind randomized controlled trial. Burns.

[23] Ibrahim Z.M., Ali O.I., Moawd S.A., Eid M.M., Taha M.M. (2021). Low Vibrational Training as an Additional Intervention for Postural Balance, Balance Confidence and Functional Mobility in Type 2 Diabetic Patients with Lower Limb Burn Injury: A Randomized Clinical Trial. Diabetes Metab. Syndr. Obes..

[24] Amtmann D., McMullen K., Bamer A., Fauerbach J.A., Gibran N.S., Herndon D., Schneider J.C., Kowalske K., Holavanahalli R., Miller A.C. (2020). National Institute on Disability, Independent Living, and Rehabilitation Research Burn Model System: Review of Program and Database. Arch. Phys. Med. Rehabil..

[25] Ewing J.A. (1984). Detecting Alcoholism: The CAGE Questionnaire. JAMA.

[26] Bamer A.M., McMullen K., Gibran N., Holavanahalli R., Schneider J.C., Carrougher G.J., Wiechman S., Wolfe A., Amtmann D. (2020). Factors Associated with Attrition of Adult Participants in a Longitudinal Database: A National Institute on Disability, Independent Living, and Rehabilitation Research Burn Model System Study. J. Burn Care Res..

[27] StataCorp (2017). Stata Statistical Software: Release 15.1.

[28] Değer T.B., Saraç Z.F., Savaş E.S., Akçiçek S.F. (2019). The Relationship of Balance Disorders with Falling, the Effect of Health Problems, and Social Life on Postural Balance in the Elderly Living in a District in Turkey. Geriatrics.

[29] Jia H., Lubetkin E.I., DeMichele K., Stark D.S., Zack M.M., Thompson W.W. (2019). Prevalence, risk factors, and burden of disease for falls and balance or walking problems among older adults in the U.S. Prev. Med..

[30] Mitchell M.B., Bhattacharyya N. (2023). Balance Disorder Trends in US Adults 2008–2016: Epidemiology and Functional Impact. OTO Open.

[31] WHO Ageing and Health, World Health Organization Published 1 October 2022. https://www.who.int/news-room/fact-sheets/detail/ageing-and-health.

[32] Tompkins R.G. (2015). Survival from burns in the new millennium: 70 years’ experience from a single institution. Ann. Surg..

[33] Chen M., Bolt G., Hooimeijer P. (2024). The impact of residential environment on older people’s capabilities to live independently: A survey in Beijing. BMC Public Health.

[34] Office of Disease Prevention and Health Promotion Social Determinants of Health. Healthy People 2030. Published 2023. https://health.gov/healthypeople/priority-areas/social-determinants-health.

[35] Hergenrather K.C., Zeglin R.J., McGuire-Kuletz M., Rhodes S.D. (2015). Employment as a Social Determinant of Health: A Systematic Review of Longitudinal Studies Exploring the Relationship Between Employment Status and Physical Health. Rehabil. Res. Policy Educ..

[36] Ross C.E., Mirowsky J. (1995). Does employment affect health?. J. Health Soc. Behav..

[37] Appelhans B.M., Gabriel K.P., Lange-Maia B.S., Karavolos K., Ylitalo K.R., Karvonen-Gutierrez C.A., Kravitz H.M., Janssen I. (2022). Longitudinal associations of mid-life employment status with impaired physical function in the Study of Women’s Health Across the Nation. Ann. Epidemiol..

[38] Takahashi P.Y., Ryu E., Jenkins G.D., Yost K.J., Kirt C.R., Larson N.L., Gupta R., Cerhan J.R., Olson J.E. (2022). Employment Characteristics and Risk of Hospitalization Among Older Adults Participating in the Mayo Clinic Biobank. Mayo. Clin. Proc. Innov. Qual. Outcomes.

[39] Carrougher G.J., Bamer A.M., Mandell S.P., Brych S., Schneider J.C., Ryan C.M., Kowalske K., Esselman P.C., Gibran N.S. (2020). Factors Affecting Employment After Burn Injury in the United States: A Burn Model System National Database Investigation. Arch. Phys. Med. Rehabil..

[40] Hutter M.F., Smolle C., Kamolz L.P. (2022). Life after Burn, Part I: Health-Related Quality of Life, Employment and Life Satisfaction. Medicina.

[41] Dyster-Aas J., Kildal M., Willebrand M. (2007). Return to work and health-related quality of life after burn injury. J. Rehabil. Med..

[42] Lampiasi N., Jacobs M. (2010). The role of physical and occupational therapies in fall prevention and management in the home setting. Care Manag. J..

[43] Whitney S.L., Marchetti G.F., Ellis J.L., Otis L. (2013). Improvements in balance in older adults engaged in a specialized home care falls prevention program. J. Geriatr. Phys. Ther..

[44] Wolf B., Feys H., De Weerdt van der Meer J., Noom M., Aufdemkampe G., Noom M. (2001). Effect of a physical therapeutic intervention for balance problems in the elderly: A single-blind, randomized, controlled multicentre trial. Clin. Rehabil..

[45] Lezotte D.C., Hills R.A., Heltshe S.L., Holavanahalli R.K., Fauerbach J.A., Blakeney P., Klein M.B., Engrav L.H. (2007). Assets and liabilities of the Burn Model System data model: A comparison with the National Burn Registry. Arch. Phys. Med. Rehabil..

[46] Wolfe A.E., Stockly O.R., Abouzeid C., Rodríguez-Mercedes S.L., Flores L.E., Carrougher G.J., Gibran N.S., Holavanahalli R., McMullen K., Trinh N.H. (2022). Burn model system national longitudinal database representativeness by race, ethnicity, gender, and age. PM R.

